# A combined reference panel from the 1000 Genomes and UK10K projects improved rare variant imputation in European and Chinese samples

**DOI:** 10.1038/srep39313

**Published:** 2016-12-22

**Authors:** Wen-Chi Chou, Hou-Feng Zheng, Chia-Ho Cheng, Han Yan, Li Wang, Fang Han, J. Brent Richards, David Karasik, Douglas P. Kiel, Yi-Hsiang Hsu

**Affiliations:** 1 Institute for Aging Research, Hebrew SeniorLife, Department of Medicine, Beth Israel Deaconess Medical Center and Harvard Medical School, Boston, MA, USA; 2 Broad Institute of MIT and Harvard, Cambridge, MA, USA; 3 Institute of Aging Research, School of Medicine, Hangzhou Normal University, and the Affiliated Hospital of Hangzhou Normal University, Hangzhou, Zhejiang, China; 4 Department of Pulmonary, Critical Care Medicine, Peking University People’s Hospital, Beijing, China; 5 Department of Medicine, Human Genetics, Epidemiology and Biostatistics, Lady Davis Institute for Medical Research, Jewish General Hospital, McGill University, Montreal, Quebec, Canada; 6 Twin Research and Genetic Epidemiology, King’s College London, London, United Kingdom; 7 Molecular and Integrative Physiological Sciences, Harvard School of Public Health, Boston, MA, USA

## Abstract

Imputation using the 1000 Genomes haplotype reference panel has been widely adapted to estimate genotypes in genome wide association studies. To evaluate imputation quality with a relatively larger reference panel and a reference panel composed of different ethnic populations, we conducted imputations in the Framingham Heart Study and the North Chinese Study using a combined reference panel from the 1000 Genomes (N = 1,092) and UK10K (N = 3,781) projects. For rare variants with 0.01% < MAF ≤ 0.5%, imputation in the Framingham Heart Study with the combined reference panel increased well-imputed genotypes (with imputation quality score ≥0.4) from 62.9% to 76.1% when compared to imputation with the 1000 Genomes. For the North Chinese samples, imputation of rare variants with 0.01% < MAF ≤ 0.5% with the combined reference panel increased well-imputed genotypes by from 49.8% to 61.8%. The predominant European ancestry of the UK10K and the combined reference panels may explain why there was less of an increase in imputation success in the North Chinese samples. Our results underscore the importance and potential of larger reference panels to impute rare variants, while recognizing that increasing ethnic specific variants in reference panels may result in better imputation for genotypes in some ethnic groups.

Computational imputation based on linkage disequilibrium (LD) patterns of phased haplotypes in a reference sample provides an alternative approach to *in*-*silico* genotype a SNP without actual genotyping^1^. Imputation of non-genotyped SNPs in multiple cohort studies with genotypes from different SNP arrays have improved statistical power by combing test statistics from both imputed and genotyped SNPs across studies^2^. This approach has become a routine practice for common SNPs in most of the genome-wide association studies. Two imputation reference panels are commonly used, including reference panels from the International HapMap Project^3^ and 1000 Genomes (1000 G) Project^4^,^5^,^6^. The 1000 G reference panel is popular due to the deeper coverage of the human genome based on 1,092 whole genome sequences in multiple ethnic samples^7^. Simulation studies have found that increasing sample size in the reference panel may improve imputation accuracy, especially for SNPs with relatively low minor allele frequencies^8^. A large reference panel may capture many less-common and rare variants, which should provide a better resolution to establish haplotype background for observed variants^9^,^10^,^11^,^12^,^13^,^14^,^15^.

In the current study, we used a relatively new reference panel with 4,873 whole genome sequences (1,092 from 1000 G and 3,781 from UK10K Project). We evaluated the imputation accuracy for both common and rare SNPs in a large-scale Caucasian Cohort study, the Framingham Heart Study (FHS), by comparing the imputed genotypes and actual genotypes from SNP arrays. In addition, previous studies also demonstrated that using ethnicity-specific reference panels to impute genotypes of the targeted samples with the same ancestral genetic background might improve imputation accuracy especially for those ethnic-specific variants^14^. The improvement of imputation accuracy is due to the fact that LD patterns of the ethnic-specific variants may not be captured by other ethnic groups with different ancestral genetic background. However, for the SNPs that are not ethnic-specific, using a reference panel from other ethnic groups or mixed ethnic groups may still provide accurate imputation. To evaluate this hypothesis, we performed whole genome imputation to generate un-genotyped variants in an East Asian cohort, North Chinese Study (NCS), using our new reference panel (1000 G + UK10K, with a majority of sequences from samples of European-descent) and estimated imputation quality of SNPs grouped into different ancestral-distance between East Asians and Europeans estimated by population differentiation score (fixation index, “F_ST_”).

## Results

### Genotype imputation of FHS data

We performed whole genome imputation in 8,700 FHS participants with 536,217 genome-wide genotypes from Affymetrix 550 K SNP-chips. The imputations with 1000 G and the combined 1000 G + UK10K generated 29,605,432 and 39,849,120 variants including the input genotypes (Table 1). There were 33% more variants generated with the 1000 G + UK10K panel than with the 1000 G panel. The imputations with 1000 G and 1000 G + UK10k added 15,245,172 and 21,449,101 variants to 105,796 low-frequency (MAF 1–5%) and rare (MAF 0.01–1%) actual genotypes, and added 8,068,486 and 11,639,300 variants to 1,578 actual genotypes with MAF less than 0.01%. Among imputed variants, 65.5% and 67.6% were variants with 0 < MAF ≤ 1% when imputed from the 1000 G and the 1000 G + UK10K panels respectively. The most abundant imputed variants were the variants with 0.01% < MAF ≤ 0.5%. The complete numbers of actual genotypes and imputed variants stratified by MAFs are listed in Table 1.

### Quality of FHS imputation

**Figure 1:**
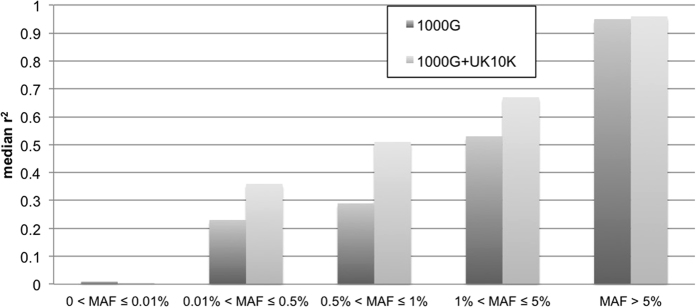
Imputation quality of FHS data evaluated by squared correlation (R^2^) between actual allelic dosages and imputed allelic dosages from imputations with 1000 G and 1000 G + UK10K. The actual allelic dosages were from a second set of FHS genotype data, OMNI5, and the original genotypes (Affy550K, the input genotype data) were excluded. The MAFs were estimated from variants imputed with 1000 G + UK10K reference panel.

We evaluated the quality of the FHS imputation by median squared correlations (r^2^) between actual allelic dosages from the OMNI5 chip and allelic dosages from imputations with 1000 G and 1000 G + UK10K. Figure 1 displays the r^2^ of 2,752,953 variants within categories of MAF estimated using the 1000 G + UK10K imputation results. The median r^2^ of imputation with 1000 G + UK10K was higher than the median r^2^ of 1000 G in most of MAF ranges except the very rare alleles (0 < MAF ≤ 0.01%), but the median r^2^ in the very rare MAF range was very low, e.g. 0.01 and 0 in 1000 G and 1000 G + UK10K imputations. The median r^2^ for SNPs with MAF > 5% were 0.95 and 0.96 in imputations of 1000 G and 1000 G + UK10K, respectively. Imputation using the 1000 G + UK10K had the greatest r^2^ improvement of 0.22 for variants with MAF 0.5–1%, while variants with MAF 0.01–0.5% had 0.13 increase in the median r^2^. Figure S1 shows a table with detailed breakdown of FHS imputation quality.

We also evaluated imputation quality when excluding SNPs with MAF ≤ 2% from the measured genotypes. The median r^2^ of imputation with 1000 G reference panel were 0.16, 0.27, and 0.4 within MAF ranges 0.01–0.5%, 0.5–1%, and 1–2%, respectively. The median r^2^ of imputation with 1000 G + UK10K reference panel were 0.29, 0.48, and 0.57 within the same MAF ranges. The detailed results are shown in Supplementary Table S3. In addition, we also performed imputations by using DuoHMM to take into account family relations in FHS samples. With or without family information, the median r^2^ of 1000 G + UK10K imputation both improved by 0.19 for variants of MAF 1–2%. Family information slightly improved 1000 G + UK10K imputation from 0.57 to 0.64. The results are shown in Supplementary Table S4.

### Increased proportion of well-imputed variants in FHS imputation with 1000 G + UK10K reference panel

We compared proportions of well-imputed variants across categories of imputation quality scores (INFO score, estimated by IMPUTE2) in all MAF ranges. Since most GWAS analyses commonly exclude low quality imputed variants with INFO scores lower than 0.4, we used INFO scores of 0.4, 0.5, 0.6, 0.7, 0.8 and 0.9 to evaluate proportions of well-imputed variants. As shown in Table 2, overall, imputation with 1000 G + UK10K had a larger proportion (increased from 0.4% to 27.5%) of well-imputed variants across all INFO and MAF ranges. Imputed variants with INFO score ≥0.4 and MAF > 0.5% all had well-imputed variants over 90%. Among imputed variants with INFO score ≥0.4 and MAF 0.01–0.5%, imputation with 1000 G + UK10K produced 76.1% well-imputed variants, which represented a greater increase (13.2%) compared to imputation with 1000 G in the same INFO range (62.9%). Imputed variants with INFO score ≥0.4 and 0 < MAF ≤ 0.01% had a 9.1% increase in well-imputed variants. Although imputed variants with INFO score ≥0.4 and MAF > 5% had only a 0.4% increase in well-imputed variants, their well-imputed variants were both over 99%.

Across all INFO and MAF ranges, the largest increase in well-imputed variants was 27.5% in the category of INFO score ≥0.6 and MAF 0.5–1%. Among imputed variants with INFO score ≥0.7, there was a 25.4% increase in well-imputed variants with MAFs 0.5–1%. For the imputed variants with INFO score ≥0.7 and MAF 0.5–1%, 64.2% were well-imputed with 1000 G + UK10K panel, and only 38.8% were well-imputed with 1000 G. If we used a more stringent INFO score cutoff of 0.8, variants imputed using the 1000 G + UK10K had 16.9% and 16.8% increases in MAF ranges of 0.5–1% and 1–5%. In the most stringent INFO score of 0.9, variants with MAF 1–5% had 11.9% increase compared to imputation with 1000 G. In the most rare MAF range of 0 < MAF ≤ 0.01%, if we used INFO ≥0.7, 0.8, and 0.9, imputation with 1000 G + UK10K had 3.6% to 11.6% well-imputed variants, but imputation with 1000 G did not produce any well-imputed variants.

### Distribution of the well-imputed variants with functional annotation

We investigated the proportion of 1000 G + UK10K well-imputed (INFO score ≥0.4) variants according to their functional roles. The well-imputed variants were classified using ANNOVAR^16^ with human genome version hg19. The classes of variants included those in exons, introns, non-coding RNA and intergenic regions. Table 3 shows the proportions of well-imputed variants of different types stratified by MAF. The proportion was the number of imputed variants with INFO ≥0.4 divided by total number of imputed variants. In general, the distributions of well-imputed variants were similar regardless of the assigned functional roles. The variants with MAF greater than 0.5% had a relatively large percentage of well-imputed variants, and the well-imputed proportions were at least 92.7% even for variants in non-coding and intergenic regions. The variants with MAF less than 0.5% had lower proportions of well-imputed variants. Almost all variants annotated as exonic splicing were well-imputed even in the very rare MAF range. Except exonic splicing, the well-imputed variants were evenly distributed in all MAF ranges.

### Genotype imputation of NCS data

We performed whole genome imputation in 3,042 NCS participants with 527,984 genome-wide genotypes from Affymetrix Axiom CHB 1 (657 K) array. The imputations with 1000 G and 1000 G + UK10K generated 29,198,055 and 40,611,832 variants including the input genotypes (Table 4). The imputation with 1000 G + UK10K had 39% more variants comparing to the imputation with 1000 G only. The imputations with 1000 G and 1000 G + UK10k added 14,040,190 and 16,151,464 variants to 16,173 low-frequency (MAF 1–5%) and rare (MAF 0.01–1%) actual genotypes, and added 9,753,722 and 18,276,702 variants to none actual genotype with MAF less than 0.01%. Among imputed variants, 68% and 66.9% were variants with 0 < MAF ≤ 1% when imputed from the 1000 G and the 1000 G + UK10K panels respectively. The most abundant imputed variants with 1000 G and 1000 G + UK10K were variants with MAF 0.01–0.5% and MAF 0–0.01%, respectively. The complete numbers of actual genotypes and imputed variants stratified by MAFs are listed in Table 4.

### Quality of NCS imputation

**Figure 2:**
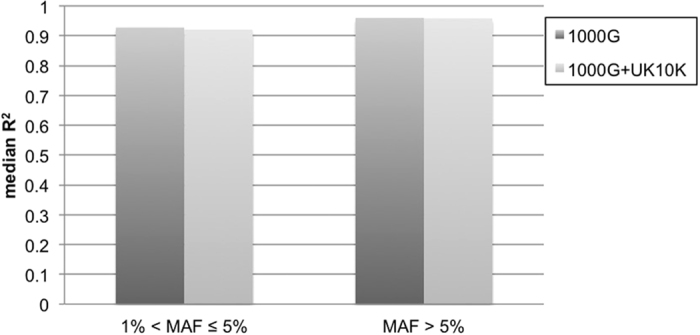
Imputation quality of NCS data evaluated by squared correlation (R^2^) between actual allelic dosages and imputed allelic dosages from imputations with 1000 G and 1000 G + UK10K. The actual allelic dosages were from the input genotype data, and the actual genotypes were first masked and then imputed to get imputed allelic dosages. The MAFs were estimated from variants imputed with 1000 G + UK10K.

Due to lack of a second source of genotype data to evaluate imputation quality in Chinese sample, we used squared correlation (R^2^) between actual allelic dosages and imputed allelic dosages by treating the actual genotypes as unknown genotypes. We stratified the R^2^ of 514,707 variants with MAFs estimated in 1000 G + UK10K imputation results in Fig. 2. We did not observe increased median R^2^ when performing imputation with 1000 G + UK10K. The median R^2^ were all above 0.92 among the low-frequent and common variants.

We then evaluated imputation quality of rare variants (MAF < 1%) by median squared correlation (r^2^, different from R^2^ that provided by IMPUTE2) between actual allelic dosages and imputed dosages, 335 SNPs with MAF < 1% (which were excluded before imputation). We observed imputation with 1000 G + UK10K decreased imputation quality by 0.19 in terms of median r^2^. The median r^2^ of imputation with 1000 G reference panel is 0.51 while the median r^2^ of imputation with 1000 G + UK10K reference panel is 0.32.

### Increased proportion of well-imputed variants in NCS imputation with 1000 G + UK10K reference panel

We compared the proportions of well-imputed variants across categories of imputation quality in a similar fashion to the FHS imputation above. We used INFO scores of 0.4, 0.5, 0.6, 0.7, 0.8 and 0.9 to evaluate proportions of well-imputed variants. As shown in Table 5, overall, imputation with 1000 G + UK10K had a larger proportion (increased from 0.2% to 14%) of well-imputed variants across all INFO and MAF ranges. Imputed variants with INFO score ≥0.4 and MAF > 0.5% all had well-imputed variants over 87%. Among imputed variants with INFO score ≥0.4 and MAF 0.01–0.5%, imputation with 1000 G + UK10K produced 61.8% well-imputed variants, which was the largest increase (12%) compared to imputation with 1000 G in the INFO range (49.8%). Imputed variants with INFO score ≥0.4 and 0 < MAF ≤ 0.01% had a 0.8% increase in well-imputed variants. Although imputed variants with INFO score ≥0.4 and MAF > 5% had only a 0.4% increase in well-imputed variants, their well-imputed variants are both over 99.5%.

Across all INFO and MAF ranges, the largest increase in well-imputed variants was 14% in the category of INFO score ≥0.5 and MAF 0.01–0.5%. Among imputed variants with INFO score ≥0.6, there was a 12.7% increase in well-imputed variants with MAFs 0.5–1%. For the imputed variants with INFO score ≥0.6 and MAF 0.5–1%, 60.3% were well-imputed with 1000 G + UK10K panel, and only 47.6% were well-imputed with 1000 G. If we used a more stringent INFO score cutoff of 0.8, variants imputed using the 1000 G + UK10K had 5.8% increases in MAF ranges of 1–5%. In the most stringent INFO score of 0.9, variants with MAF 1–5% had 4% increase compared to imputation with 1000 G. In the most rare MAF range of 0 < MAF ≤ 0.01%, both imputations with 1000 G and 1000 G + UK10K had less than 1% well-imputed variants even when we used the most loosen INFO of 0.4.

### Imputation quality and Population Difference Estimated by fixation index (F_ST_)

**Figure 3:**
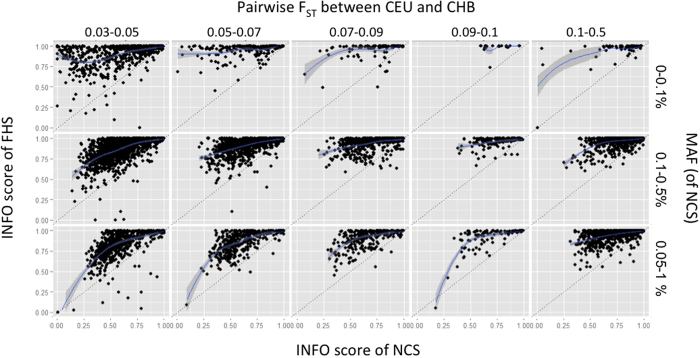
Correlation between the FHS and NCS imputation results, by MAF and F_ST_. The x and y axes of each small plot are INFO scores of FHS and NCS. Small plots show distribution of two INFO scores within a range of F_ST_ and MAF. CEU and CHB stand for Utah residents with ancestry from Northern and Western Europe and Han Chinese in Beijing.

Compared to imputation results in the FHS, we observed smaller increases in well-imputed rare variants in NCS imputation results. The proportion of well-imputed variants with MAF 0.1–0.5% increased by 21% in the FHS imputation in contrast to only a 9% increase in NCS when imputations were done using the 1000 G + UK10K panel. We first checked the population structure of all individuals by principal component analysis (PCA). In the PCA result shown in Fig. S2, FHS data overlapped well with the 1000 G European and UK10K data, and the NCS data overlapped with the 1000 G Asian data. F_ST_, which describes the population differentiation due to genetic structure, was then calculated for 14,339,925 variants existing both in FHS and NCS imputation results to investigate the relationship between imputation quality and F_ST_. Figure 3 shows the INFO score of imputed variants in both the FHS and NCS cohorts. The pattern of the plots suggested a decreasing quality of the NCS imputation results (dots shifted toward left) whereas the FHS imputation results maintained good quality (dots remained above the line of identity). For example, in the plot with MAF 0.1–0.5% and F_ST_ 0.09–0.1, most imputed variants in FHS had INFO scores higher than 0.75, and imputed variants in NCS had INFO scores widely distributed (from 0.38 to higher).

#### Computing cost

Genome-wide imputation of FHS genotypes with 1000 G reference panel took 2,422 computing hours in total (with 773 parallel jobs), and imputation with 1000 G + UK10K took 4,688 computing hours (with 908 parallel jobs).

## Discussion

Our ability to comprehensively ascertain both common and rare genetic variants may bring us a step closer to understanding the genetic architecture of a disease. In addition, rare genetic variants may explain missing heritability that common variants fail to explain. Thus, identifying rare variants associated with phenotypes and diseases become a more important task when investigating genetic risk factors. Whole genome sequencing is the best way to find rare variants in the genome. However, the cost of whole genome sequencing for a significant amount of individuals at this time is still expensive, and there remains considerable interest by large-scale enterprises, such as GEFOS^17^ and UK Biobank^18^, in analyzing imputed genotypes. Our work suggests that increasing sample size of the reference panel increases imputation quality, especially for rare variants. Huang *et al*. recently demonstrated the same finding when comparing imputation results between 1000 G and 1000 G + UK10K^19^. In addition, our findings also suggest that even if the reference panel is not ethnic-specific for the targeted samples, the imputation quality is still improved. Comparing the improvement of the imputation quality between Chinese samples and Caucasian samples by using 1000 G + UK10K reference panel, we found that the improvement in imputation quality of Chinese samples was not as good as in the Caucasian samples. This was not surprising since the 1000 G + UK10K reference panel has an increased proportion of similar (Caucasian) ethnic samples (adding 3,000 Caucasian samples from the UK10K project). We suggest that to increase imputation quality, the best strategy is to use reference panels with the specific ethnic composition of the study population being imputed as this may improve the imputation of relatively rare SNPs.

In this study, we investigated genotype imputation improvement in terms of quality and quantity especially on rare variants with the 1000 G + UK10K reference panel in Caucasian and Chinese cohorts. The imputations with 1000 G + UK10K reference added 9,783,688 and 11,413,777 additional variants in total to the imputation using the 1000 G reference panel alone in FHS and NCS, respectively. Our findings suggested that a larger reference panel improved imputation performance, especially for less-common and rare variants, even for Chinese samples. On the other hand, in the FHS, the imputation quality for common variants (MAF > 5%) only improved by 0.01 according to the r^2^ estimated between actual allelic dosages and imputed allelic dosages. One potential explanation for this finding is that using the 1000 G as a reference panel already provides high quality imputation (r^2^ = 0.95) for common variants. In NCS imputation, for common variants, we did not see much overall improvement in quality in the imputation with the 1000 G + UK10K reference panel. Our observation confirms a recent study that demonstrated the value of study-specific reference panels^20^. However, even when using INFO score ≥0.4 as cutoff, the 1000 G + UK10K panel still increased the fraction of well-imputed variants in NCS samples. Number of well-imputed SNPs (with INFO score ≥0.4, using 1000 G + UK10K reference) with MAF 0.01–0.5% increased from 62.9% to 76.1% and 49.8% to 61.8% in FHS and NCS, respectively. We still observed increased proportions of well-imputed variants when we used more stringent INFO score cutoff.

We used variants’ F_ST_ scores representing the population differentiation between Caucasian and Chinese to explain why the imputation of NCS samples did not have comparable qualities to the imputation of FHS samples based on 1000 G + UK10K reference panel. Our findings suggest that some rare variants are commonly existing in both ethnicities and these variants may retain haplotype structure across ethnicities which allow us to impute some rare variants with high quality given the underlying genetic background is different between reference panel and targeted samples.

There are potential limitations in this study. First, we compared imputation quality between two different-ethnicity samples, Chinese and Caucasian. However, the difference between these two samples include sample size, genotype platform and density of SNPs, which may all affect imputation quality, in addition to the differences in genetic background. Our results may not be applicable to other ethnic groups, such as African populations due to their short haplotype blocks compared to Caucasian and East Asian populations. Second, major imputation results of family-based FHS were not performed using family information. However, results of a small set FHS imputation with family information did not change our conclusion on imputation improvement with 1000 G + UK10K reference panel. Third, this study only used genotyping data generated from Affymetrix chips to impute genotypes. We cannot estimate the influences that may possibly come from different density of variants or including rare variants in their genotyping platforms. We thus tested a small set of FHS imputation excluding variants with MAF ≤ 2% in the input measured genotypes. The imputation quality without rare variants was consistent with the findings when actual genotypes with MAF ≤ 2% were included. For 335 rare variants of Chinese samples (with MAF < 1%), we observed decreased imputation quality when 1000 G + UK10K were used. More data on Chinese-specific rare variants are needed to confirm this source for decrease in quality. We didn’t evaluate whether even larger reference panels such as the human haplotypes provided by the Haplotype Reference Consortium (HRC, http://www.haplotype-reference-consortium.org/), will improve a lot of the imputation quality of the relatively rare variants. The HRC was not yet available when we performed our analyses. HRC panel consists of 64,976 haplotypes with 39,235,157 SNPs. Majority of the HRC references are Caucasian samples, thus, we expect such larger reference panel may further increase imputation quality for the rare variants in Caucasian samples, but may not necessarily improve imputation quality of non-Caucasian samples.

Although the imputation quality for rare variants improved in our study, still, more than half of the rare variants were not imputed with considerable high quality (having INFO score < 0.4); thus, these variants cannot be used in association analyses. It will be necessary to estimate how large the sample size of the reference panel should be, in order to have more than 80% of imputed rare variants with INFO score ≥0.4. We were also not able to evaluate the effect of sample size in the targeted samples to the imputation quality.

In summary, we evaluated imputation quality by imputing un-genotyped SNPs in a Caucasian sample and a North Chinese sample using a combined reference panel (N = 3781) by combing whole genome sequences from 1000 G and UK10K projects. We found that our proposed reference panel outperformed the 1000 G reference panel, especially for relatively rare variants in the Caucasian sample, suggesting that increasing sample size in a reference panel, especially ethnic specific sample size (the same ethnicity of the targeted sample) would improve imputation quality. Nevertheless, the imputation quality was still improved in common variants for non ethnic-specific samples (for example, imputation on North Chinese samples in current study) although the improvement is limited.

## Methods

### Reference panel: 1000 Genome project (1000 G)

The publicly available database of the Phase 1 version 3 of the 1000 G contains 1,092 individuals including the following populations: 181 Admixed American, 246 African, 286 East Asian, and 379 European^7^. The data were downloaded from the IMPUTE website (ALL_1000 G_phase1integrated_v3_impute_macGT1.tgz, build date: 2012-08-25). In the 1000 G data set, monomorphics, singleton and doubleton single nucleotide variants (SNVs with minor allele count less than two) were removed. A total of 30,061,896 SNVs were used for imputations.

### Reference panel: UK10K project (UK10K)

The database from the UK10K project had a total of 3,781 individuals who underwent whole genome sequencing from the Avon Longitudinal Study of Parents and Children (ALSPAC) and the King’s College London Department of Twin Research and Genetic Epidemiology Twins Registry (TWINSUK). The UK10K data (IDs: EGAD00001000195 and EGAD00001000194) were downloaded from the European Genome-Phenome Archive. The two data sets were first merge by PLINK^21^ and then haplotype phased by SHAPEIT2^22^. We excluded monomorphics, singleton and doubleton single nucleotide variants (SNVs with minor allele count less than two) from the UK10K data set. A total of 27,793,639 SNVs were used for imputations.

### Reference panel: The combined reference panel of 1000 G and UK10K (1000 G + UK10K)

We used IMPUTE2^23^ to merge 1000 G and UK10K reference panels and generate a combined reference panel. There are 47,151,447 SNVs from 4,873 individuals in the merged 1000 G + UK10K reference panel. This combined reference panel was used to impute genotypes. Table S1 shows the three reference panels.

### Study Sample and Genotyping of Framingham Heart Study (FHS)

The FHS contains family members and unrelated participants. The participants are Caucasian of European descent. Two sets of genotyping data, Affy550K and OMNI5 were generated by the FHS SHARe (SNP Health Association Resource) project. The first set, Affy550K, with 549,827 SNPs (Affymetrix 500 K mapping array plus Affymetrix 50 K supplemental array) came from genotyping in over 9,274 FHS subjects from over 900 families^24^. By estimation, we expected at least 80% genomic coverage (pair-wise genotype correlation *r*^2^ > 0.8) of the HapMap Phase I + II common SNPs (minor allele frequency, MAF ≥ 0.05) for the Caucasian population^25^. We re-confirmed the kinship relationship among the Framingham pedigrees using all available SNPs. PEDCHECK^26^ and RELPAIR^27^ programs were used to check for errors in Mendelian inheritance and inconsistencies within pedigrees. We excluded 793 individuals with an average SNP call rate of <0.97. We also excluded individual SNPs with a call rate of <0.95 (34,868 SNPs; 6.3%); Hardy-Weinberg equilibrium (HWE) test p-value < 1 × 10^−6^ (8,531 SNPs; 1.6%); MAF < 0.01 (66,829 SNPs; 12.2%); or unknown genomic annotation (6,089 SNPs; 1.1%). After quality control, 455,458 total SNPs remained for analysis. Among the SNPs, 367,873 (80.8%) had MAF > 5%, 47,116 (10.3%) had 1% < MAF ≤ 5%, 8,728 (1.9%) had 0.5% < MAF ≤ 1%, 16,932 (3.7%) had 0.1% < MAF ≤ 0.5%, and 14,809 (3.3%) had 0 < MAF ≤ 0.1%.

The second source of FHS genotype data, OMNI5, was used to validate imputation results. The OMNI5 data were generated using the Illumina OMNI5 bead chip, and included 4,271,233 SNPs genotyped in 2,474 FHS subjects. We excluded one individual with an average SNP call rate of <0.97. We also excluded individual SNPs with a call rate of <0.95; HWE test p-value < 1 × 10^−6^. Among the 3,478,371 remaining SNPs from 2,472 individuals, 1,661,955 (47.8%) had MAF > 5%; 814,798 (23.4%) had 1% < MAF ≤ 5%; 292,077 (8.4%) had 0.5% < MAF ≤ 1%; 364,555 (10.5%) had 0.1% < MAF ≤ 0.5%; and 344,986 (9.9%) had 0% < MAF ≤ 0.1%.

### Study Sample and Genotyping of North Chinese Study (NCS)

The NCS contains 1,189 narcolepsy cases from North China (n = 1136), Taiwan (n = 51) and Stanford University (n = 2) and 1,997 controls from China^28^. The genotyping was conducted using the Affymetrix Axiom CHB array with 640,674 available SNPs on the chips. We excluded individuals with an average SNP call rate of <0.98. Among the remaining 3,042 individuals, we also excluded individual SNPs with a call rate of <0.95, HWE test p-value < 1 × 10^−6^, and MAF < 0.01. Among the 527,984 remaining SNPs, 511,811 (96.9%) had MAF > 5% and 16,173 (3.1%) had 1% < MAF ≤ 5%. Table S2 shows the three sets of genotype data.

### Haplotype phasing and genotype imputation tools

Input genotype data sets for imputation were first pre-phased with SHAPEIT2^22^, and then missing genotypes were imputed with IMPUTE2^2^ by 5-Mb region at a time against the 1000 G or the combined reference panel. To evaluate the effect of pedigree information to imputation, we post-process phased haplotypes with duoHMM to incorporate pedigree information.

### Estimation of overall genotype imputation quality by squared Pearson correlation (r^2^ and R^2^)

To estimate overall genotype imputation quality, we calculated squared Pearson correlation (r^2^) between actual (discrete) allelic dosage and the imputed (continuous) allelic dosage over a defined set of samples. For FHS, we excluded overlapping genotypes both existing in Affy550K and OMNI5 data, and the remaining genotypes overlapping between 1000 G and 1000 G + UK10K panels were used to examine imputation quality by r^2^. For NCS, due to lack of a second set of genotyping data, we estimated imputation quality by squared correlation (R^2^) between input actual allelic dosage and the allelic dosage imputed for the input genotypes. The imputed allelic dosages were generated with the input genotype masked. The R^2^ measurements of NCS were provided by IMPUTE2.

### Estimated imputation quality score (INFO)

IMPUTE2 uses INFO to indicate the certainty of each imputation. The INFO scores range between 0 and 1, and values near 1 indicate high imputation certainties^2^.

### Principle component analysis (PCA)

We performed PCA to find directions of the largest variability of the genotype data. We used the first two principal components to reflect the geographical distance of all individuals from reference panels and our study samples. The PCA was performed using chromosome 20.

### Population differentiation measured by fixation index (F_ST_)

We used a F_ST_ to describe the population differentiation due to genetic structure at the level of the SNP. F_ST_ of zero indicates that the SNP has no divergence between populations. To analyze the imputation results of FHS and NCS with the combined reference panel, we used pairwise F_ST_ scores between Utah Residents with Northern and Western European ancestry (CEU) and Han Chinese in Beijing (CHB) calculated by the method of Weir and Cockerham^29^. The F_ST_ scores of CEU and CHB were calculated using the 1000 G project data^30^.

## Additional Information

**How to cite this article**: Chou, W.-C. *et al*. A combined reference panel from the 1000 Genomes and UK10K projects improved rare variant imputation in European and Chinese samples. *Sci. Rep.* **6**, 39313; doi: 10.1038/srep39313 (2016).

**Publisher's note:** Springer Nature remains neutral with regard to jurisdictional claims in published maps and institutional affiliations.

## Supplementary Material

Supplementary Information

## Figures and Tables

**Table 1:** Number of actual genotypes and imputed variants in FHS.

	MAF = 0	0 < MAF ≤ 0.01%	0.01% < MAF ≤ 0.5%	0.5% < MAF ≤ 1%	1% < MAF ≤ 5%	MAF > 5%	Total
Genotyped by Affy550K chips	1,578 (0.3%)	0	30,175 (5.6%)	15,171 (2.8%)	60,450 (11.3%)	428,843 (80%)	536,217
Imputed with 1000 G	1,235,417 (4%)	6,834,647 (23%)	11,141,317 (38%)	1,410,031 (5%)	2,799,620 (9%)	6,184,400 (21%)	29,605,432
Imputed with 1000 G + UK10K	3,897,784 (10%)	7,743,094 (20%)	17,568,163 (45%)	1,296,955 (3%)	2,689,779 (7%)	6,193,345 (16%)	39,389,120

**Table 2:** Proportion of well-imputed variants in FHS imputation results.

MAF		0 < MAF ≤ 0.01%		0.01% < MAF ≤ 0.5%		0.5% < MAF ≤ 1%		1% < MAF ≤ 5%		MAF > 5%	
Reference panel		1000 G	1000 G + UK10K	1000 G	1000 G + UK10K	1000 G	1000 G + UK10K	1000 G	1000 G + UK10K	1000 G	1000 G + UK10K
Total imputed variants		6,834,647	7,743,094	11,141,317	17,568,163	1,410,031	1,296,955	2,799,620	2,689,779	6,184,400	6,193,345
INFO	≥0.4	12.0%	21.2%	62.9%	76.1%	90.8%	98.4%	97.1%	99.2%	99.3%	99.8%
	≥0.5	9.0%	17.9%	46.4%	62.1%	74.5%	94.3%	89.3%	97.7%	97.8%	99.4%
	≥0.6	4.9%	15.1%	33.0%	46.0%	55.7%	83.2%	77.3%	93.2%	95.0%	98.4%
	≥0.7	0.0%	11.6%	22.5%	31.4%	38.8%	64.2%	64.2%	83.4%	91.0%	96.4%
	≥0.8	0.0%	7.4%	13.4%	19.5%	25.0%	41.9%	51.0%	67.8%	85.0%	92.2%
	≥0.9	0.0%	3.6%	5.2%	10.1%	14.1%	23.0%	36.9%	48.8%	73.9%	83.5%

The imputations were performed with 1000 G and 1000 G + UK10K reference panels.

**Table 3:** Proportion of well-imputed variants with functional roles and MAFs.

Functional roles	0 < MAF ≤ 0.1%	0.1% < MAF ≤ 0.5%	0.5% < MAF ≤ 1%	1% < MAF ≤ 5%	MAF > 5%
Exons	78.3%	90.1%	95.7%	97.9%	99.1%
Nonsense	79.6%	87.1%	96.3%	95.8%	100%
Splicing	100%	83.3%	100%	100%	100%
Missense	78.2%	90.8%	97.0%	98.6%	99.3%
3′UTR	79.1%	91.2%	96.8%	98.7%	99.3%
5′UTR	78.7%	91.3%	95.9%	98.2%	99.4%
Non-coding RNAs located in exons	77.7%	88.1%	92.7%	96.2%	98.7%
Non-coding RNAs located in introns	80.3%	91.9%	97.3%	99.1%	99.4%
Intergenic regions	79.2%	90.9%	96.7%	98.7%	99.3%

**Table 4:** Number of actual genotypes and imputed variants in NCS.

	MAF = 0	0 < MAF ≤ 0.01%	0.01% < MAF ≤ 0.5%	0.5% < MAF ≤ 1%	1% < MAF ≤ 5%	MAF > 5%	Total
Genotyped by Affymetrix Axiom CHB 1 array	0	0	0	0	16,173 (3%)	511,811 (97%)	527,984
Imputed with 1000 G	1,642,218 (6%)	8,111,504 (28%)	10,354,147 (35%)	1,386,886 (5%)	2,299,157 (8%)	5,404,143 (19%)	29,198,055
Imputed with 1000 G + UK10K	4,791,282 (12%)	13,485,420 (33%)	12,236,827 (30%)	1,450,070 (4%)	2,464,567 (6%)	6,183,666 (15%)	40,611,832

The imputations were performed with 1000 G and 1000 G + UK10K reference panels.

**Table 5:** Proportion of well-imputed variants in NCS imputation results.

MAF		0 < MAF ≤ 0.01%		0.01% < MAF ≤ 0.5%		0.5% < MAF ≤ 1%		1% < MAF ≤ 5%		MAF > 5%	
Reference panel		1000 G	1000 G + UK10K	1000 G	1000 G + UK10K	1000 G	1000 G + UK10K	1000 G	1000 G + UK10K	1000 G	1000 G + UK10K
Total imputed variants		8,111,504	13,485,420	10,354,147	12,236,827	1,386,886	1,450,070	2,299,157	2,464,567	5,404,143	6,183,666
INFO	≥0.4	0.2%	0.9%	49.8%	61.8%	87.4%	94.0%	96.1%	98.3%	99.5%	99.8%
	≥0.5	0.0%	0.2%	33.8%	47.8%	67.9%	80.6%	86.3%	93.0%	98.3%	99.4%
	≥0.6	0.0%	0.0%	21.1%	33.2%	47.6%	60.3%	72.9%	81.7%	96.3%	98.3%
	≥0.7	0.0%	0.0%	12.3%	20.8%	31.4%	40.4%	59.8%	67.5%	93.8%	96.5%
	≥0.8	0.0%	0.0%	6.2%	11.5%	19.1%	24.5%	47.4%	53.2%	90.3%	93.6%
	≥0.9	0.0%	0.0%	2.2%	4.9%	9.3%	11.9%	33.8%	37.8%	83.3%	88.0%

The imputations were performed with 1000 G and 1000 G + UK10K reference panels.
