# Supplementary material for: A combined reference panel from the 1000 Genomes and UK10K projects improved rare variant imputation in European and Chinese samples
Source: Sci Rep. 2016 Dec 22;6:39313. doi: 10.1038/srep39313 (PMC5177868; doi:10.1038/srep39313)

Supplementary Information

A combined reference panel from the 1000 Genomes and UK10K projects improved rare variant imputation in European and Chinese samples

Wen-Chi Chou[1,6,*], Hou-Feng Zheng[2,*], Chia-Ho Cheng[1], Han Yan[3], Li Wang[2], Fang Han[3], J. Brent Richards[4,5], David Karasik[1], Douglas P. Kiel[1,6], Yi-Hsiang Hsu[1,6,7]

[1]Institute for Aging Research, Hebrew SeniorLife, Department of Medicine, Beth Israel Deaconess Medical Center and Harvard Medical School, Boston, MA

[2]Institute of Aging Research, School of Medicine, Hangzhou Normal University, and the Affiliated Hospital of Hangzhou Normal University, Hangzhou, Zhejiang, China

[3]Department of Pulmonary, Critical Care Medicine, Peking University People's Hospital, Beijing, China

[4]Department of Medicine, Human Genetics, Epidemiology and Biostatistics, Lady Davis Institute for Medical Research, Jewish General Hospital, McGill University, Montreal, Quebec, Canada

[5]Twin Research and Genetic Epidemiology, King's College London, London, United Kingdom

[6]Broad Institute of MIT and Harvard, Cambridge, MA

[7]Molecular and Integrative Physiological Sciences, Harvard School of Public Health, Boston, MA

*These authors contributed equally to this work.

Correspondence and requests for materials should be addressed to Y.-H.H. (email: YiHsiangHsu@hsl.harvard.edu)

Supplementary Table S1. Three reference panels used in this study.

Reference panel	Number of individuals	Ancestry	Number of variants
**1000G**	1,092	European, African, AdMixed American, East Asian, and South Asian	30,061,896 (~12,605,541 European)
**UK10K**	2,432	Europe	21,513,377
**Combined panel of 1000G and UK10K**	3,524	Combined populations from 1000G and UK10K	39,036,197

Supplementary Table S2. Three genotype data sets used for imputation in this study.

Cohort	Number of individuals	Ancestry	Number of variants
**Framingham Heart Study - Affy550**	8,477	European descent	550K SNPs (Affymetrix 500k + MIPS 50k)
**Framingham Heart Study - Omni5**	2,474	European descent	3,480K SNPs (HumanOmni5M-4v1 array (OMNI5))
**North Chinese Study**	3,042	Mostly East Asian of Han descent	657K SNPs (Affymetrix Axiom CHB 1 array)

Supplementary Table S3. Imputation quality with rare measured genotypes excluded. The imputation quality of FHS data (chromosome 20) was evaluated by median squared correlation between actual allelic dosages and imputed allelic dosages from imputations with the 1000G and 1000G+UK10K reference panels. The MAFs were estimated from imputation with 1000G+UK10K reference panel.

		**MAF**			
**Measured genotypes excluding MAF < 2%**	**Reference panel**	0-0.01%	0.01-0.5%	0.5-1%	1-2%
No	1000G	0	0.154	0.237	0.384
No	1000G+UK10K	0	0.307	0.485	0.574
Yes	1000G	0.005	0.155	0.272	0.398
Yes	1000G+UK10K	0	0.291	0.481	0.569

Supplementary Table S4. Imputation quality with additional adjustment for family structure using duoHMM process. The imputation quality of FHS data (chromosome 20) was evaluated by median squared correlation between actual allelic dosages and imputed allelic dosages from imputations with 1000G and 1000G+UK10K. The MAFs were estimated from imputation with the 1000G+UK10K reference panel.

		**MAF**			
**duoHMM used to take into account family relations**	**Reference panel**	0-0.01%	0.01-0.5%	0.5-1%	1-2%
No	1000G	0	0.154	0.237	0.384
No	1000G+UK10K	0	0.307	0.485	0.574
Yes	1000G	0.193	0.221	0.314	0.442
Yes	1000G+UK10K	0	0.390	0.551	0.636

Fig S1. Imputation quality of FHS data. The quality was evaluated by squared correlation between actual allelic dosages and imputed allelic dosages from imputations with the 1000G and 1000G+UK10K reference panels. Each small boxplot shows r^2 on the y-axis in range of MAF show at the top of the small boxplot. The red diamond indicates the average r^2.

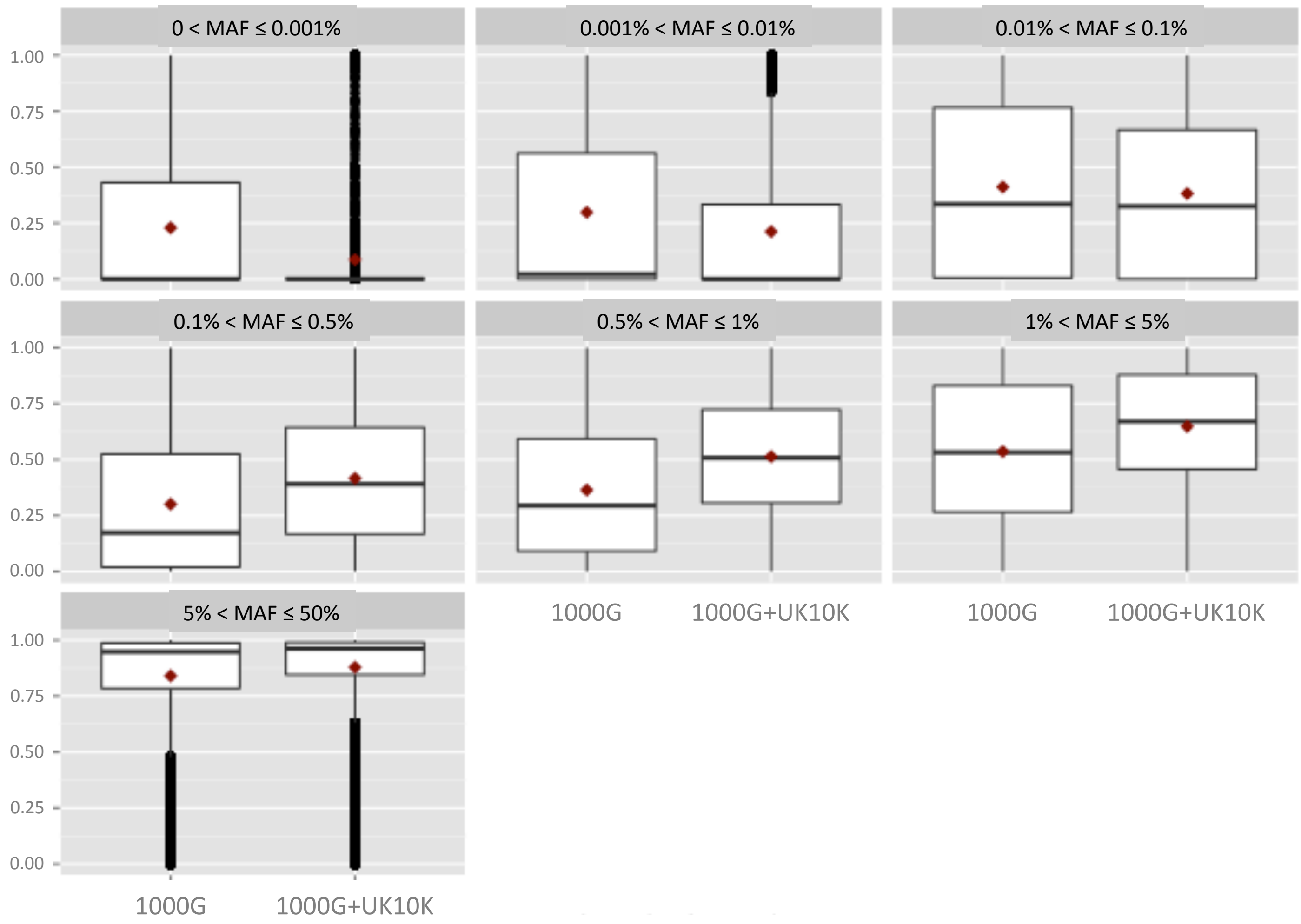

Figure S2. The PCA result of all samples including 1000G, UK10K, FHS, and NCS. X-axis denotes the value of PC1, and y-axis denotes the value of PC2, with each dot representing one individual. The shape and color for individuals from 1000G African, 1000G American, 1000G European, 1000G Asian, UK10K, FHS, and NCS are black circle, red circle, blue circle, green circle, purple square, gray diamond, and cyan triangle.

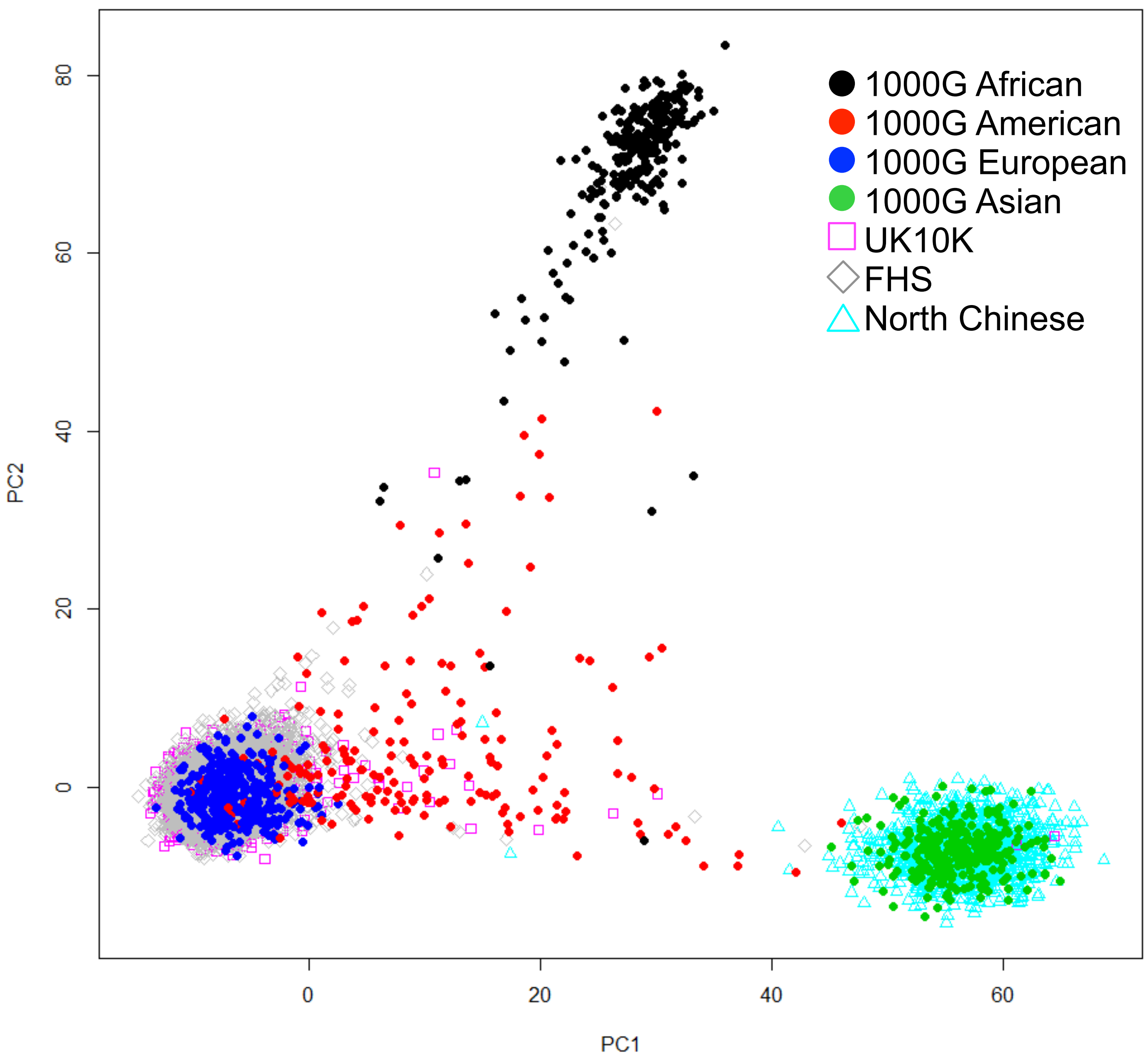

Supplement: Supplementary Information [file srep39313-s1.pdf]
